# Role of Monoubiquitylation on the Control of IκBα Degradation and NF-κB Activity

**DOI:** 10.1371/journal.pone.0025397

**Published:** 2011-10-12

**Authors:** Elisa Da Silva-Ferrada, Mónica Torres-Ramos, Fabienne Aillet, Michela Campagna, Carlos Matute, Carmen Rivas, Manuel S. Rodríguez, Valérie Lang

**Affiliations:** 1 Proteomics Unit, CIC bioGUNE, CIBERehd, Bizkaia Technology Park, Derio, Spain; 2 Centro Nacional de Biotecnología, CSIC, Campus Universidad Autónoma de Madrid, Madrid, Spain; 3 Department of Neuroscience University of the Basque Country UPV/EHU, CIBERNED, Leioa, Bizkaia, Spain; 4 Department of Biochemistry University of the Basque Country UPV/EHU, Leioa, Bizkaia, Spain; Massachusetts General Hospital, United States of America

## Abstract

The NF-κB pathway is regulated by multiple post-translational modifications including phosphorylation, ubiquitylation and SUMOylation. Many of these modifications act on the natural inhibitor IκBα modulating its capacity to control signal-mediated NF-κB activity. While the canonical pathway involving the phosphorylation and polyubiquitylation of IκBα has been well characterized, the role of these post-translational modifications in the control of basal NF-κB activity has not been deeply explored. Using the recently developed Tandem-repeated Ubiquitin Binding Entities (also known as ubiquitin traps) to capture ubiquitylated proteins, we identified monoubiquitylated forms of IκBα from multiple rat organs and cell types. The identification of these forms was demonstrated through different procedures such as immunoprecipitations with specific ubiquitin antibodies or His6-Ubiquitin pull downs. Monoubiquitylated forms of IκBα are resistant to TNFα-mediated degradation and can be captured using TUBEs, even after proteasome inhibitors treatment. As it occurs for monoSUMOylation, monoubiquitylation is not dependent of the phosphorylation of IκBα on the serines 32/36 and is not optimally degraded after TNFα stimulation. A ubiquitin-IκBα fusion exhibits phosphorylation defects and resistance to TNFα mediated degradation similar to the ones observed for endogenous monoubiquitylated IκBα. The N-terminal attachment of a single ubiquitin moiety on the IκBα fusion results in a deficient binding to the IKKβ kinase and recruitment of the SCF ligase component βTrCP, promoting a negative impact on the NF-κB activity. Altogether, our results suggest the existence of a reservoir of monoubiquitylated IκBα resistant to TNFα-induced proteolysis, which is able to interact and repress DNA binding and NF-κB transcriptional activity. Such pool of IκBα may play an important role in the control of basal and signal-mediated NF-κB activity.

## Introduction

The nuclear factor κB (NF-κB) is a family of transcription factors that regulate the expression of various genes involved in inflammatory, anti-apoptotic and immune responses [1] [2]. The NF-κB pathway can be activated by many different extra cellular signals that induce multiple post-translational modifications such as phosphorylation, ubiquitylation and SUMOylation, acting at various levels of the signaling cascade [3]–[5]. As many other stimuli, the pro-inflammatory cytokine TNFα (tumor necrosis factor-alpha) ends with the activation of the IKK (IκBα Kinase) complex, composed by IKKα, IKKβ and IKKγ/NEMO [6] [7]. IKK phosphorylates the alpha inhibitor of NF-κB, IκBα, on the serines 32 and 36 and targets it for ubiquitylation at the main ubiquitylation sites, lysine 21 and 22 by a SCF (Skp, Cullin, F-box) ubiquitin ligase complex containing the beta-transducin repeat-containing protein βTrCP) [8] [9]. The presence of the *DSGXXS* motif determines the specific interaction of βTrCP with the phosphorylated Inhibitor of NF-κB alpha (IκBα), which is crucial for its ubiquitylation and posterior proteasome degradation. In contrast, the conjugation with the small ubiquitin-like modifier 1 (SUMO-1) is not dependent on the phosphorylation on the serines 32 and 36 of IκBα and has a positive impact on IκBα stability [10]. Ubiquitylation of IκBα is tightly controlled by the action of unidentified DUBs (de-ubiquitylating enzymes). Released NF-κB is then imported to the nucleus where it activates the transcription of a large number of genes including IκBα and TNF-receptor 2 [11] [2]. Newly synthesized IκBαis imported into the nucleus where it ends up with NF-κB mediated transcription by detaching it from DNA promoter sequences and favoring its export to the cytoplasm [12] [13].

In this study, the use of ubiquitin traps (TUBEs for Tandem-repeated Ubiquitin Binding Entities) [14] allowed us to identify monoubiquitylated IκBα from rat organs, as well as from different cell lines. Using *in vitro* and *ex vivo* approaches we aimed to understand the impact that a single ubiquitin moiety can have on the properties and inhibitory capacity of IκBαThe evidence presented here suggests the existence of a pool of monoubiquitylated IκBα resistant to degradation whose function might play an important role in the control of basal and signal-induced NF-κB activity.

## Results

### Presence of monoubiquitylated IκBα in organs and cell lines

**Figure 1:**
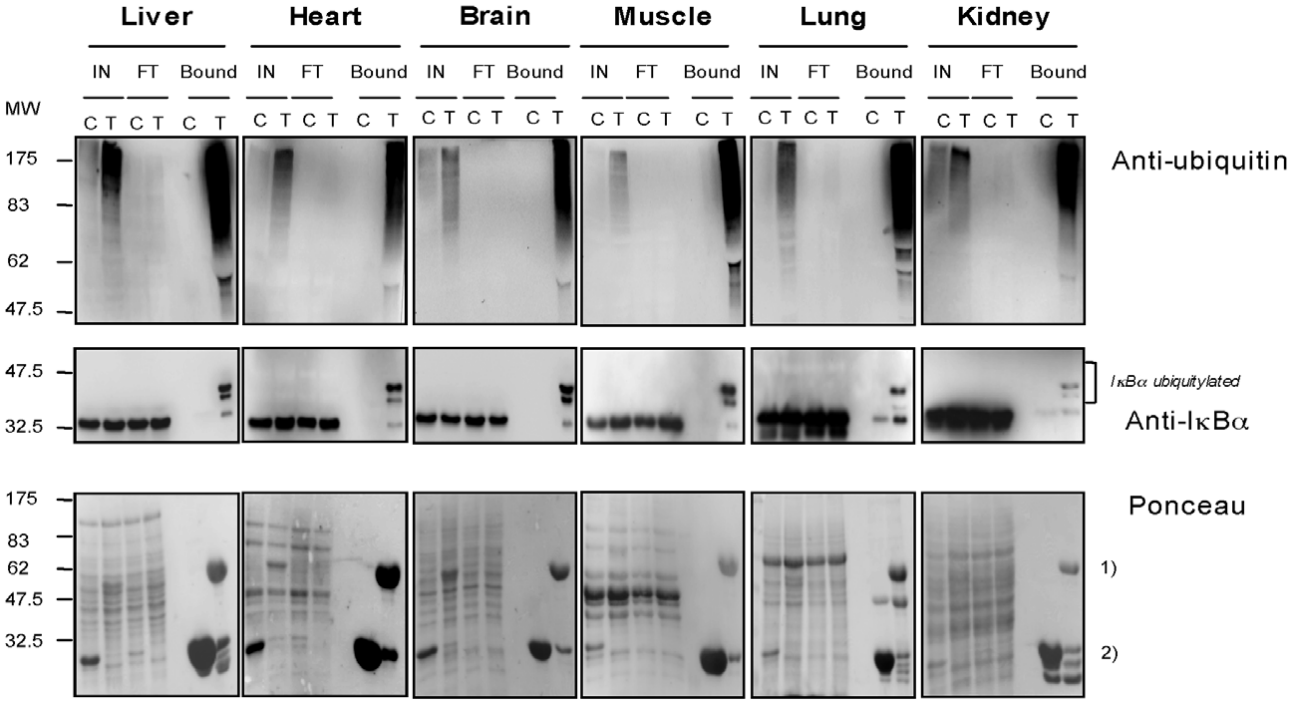
Extraction of monoubiquitylated IκBα from rat organs. Extraction of total ubiquitylated proteins from liver, brain, heart, muscle, lung and kidney [14]. TUBEs (T) or GST control (C) bound proteins were analyzed by Western blot with the anti-ubiquitin and anti-IκBα antibodies. Membranes were stained with Ponceau, used as charge control, (1) TUBEs; 2) GST). Input (IN), Flow through (FT).

**Figure 2:**
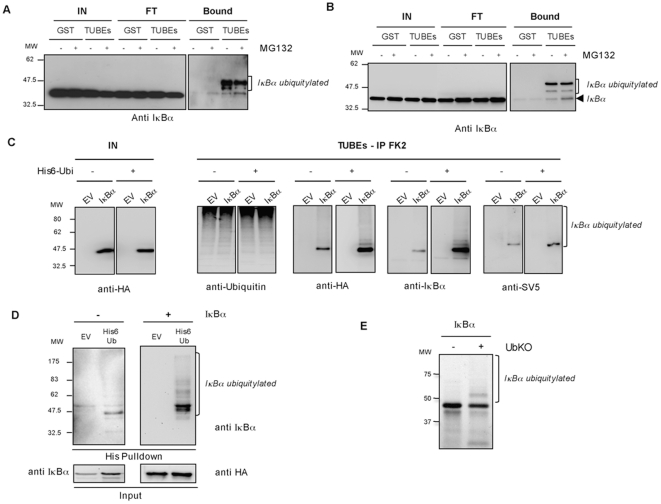
Isolation of monoubiquitylated IκBα from cell lines and *in vitro*. A, B) TUBE-based isolation and detection of monoubiquitylated IκBα from HEK293 cells (A) and Jurkat cells (B) treated or not 1 h with 20 µM of MG132. Input (IN), Flow through (FT). C) HEK293 cells were transfected with the indicated constructs before being lysed in a buffer containing 3.5 mM of TUBE hHR23A. TUBE-captured material was eluted and submitted to Ubiquitin immunoprecipitation. EV: Empty Vector. D) Nickel chromatography was used to isolate His6-Ubiquitin conjugates from HEK293 cells co-transfected (+) or not (−) with a vector expressing IκBα and His6-Ubiquitin or Empty vector (EV). E) *In vitro* ubiquitylation assay in presence of ubiquitin mutant (Ub KO) using ^35^S IκBα WT as substrate.

The recently developed ubiquitin-traps (TUBEs) that specifically capture ubiquitin and ubiquitylated proteins [14] were adapted to extract ubiquitylated proteins from rat organs. As reported, TUBEs capture preferentially polyubiquitin proteins, however monoubiquitylated proteins can also be captured when abundantly expressed [14]. Monoubiquitylated IκBα can be easily detected by Western blot in a mix of total ubiquitylated proteins purified by TUBEs from liver, heart, brain, muscle, lung and kidney rat (Figure 1), suggesting a function for this form of IκBα in normal tissues. Monoubiquitylated IκBα can also be captured using a similar procedure with multiple cell lines such as HEK293 (Figure 2A), Jurkat (Figure 2B) and HeLa (data not shown). The identification of the monoubiquitylated IκBα was confirmed using several protocols including immunoprecipitations with a specific anti-ubiquitin antibody of the TUBE-captured material. Under these conditions endogenous and exogenous monoubiquitylated IκBα can be detected using anti-IκBα, anti HA or anti SV5 antibodies, respectively (Figure 2C). Furthermore, monoubiquitylated IκBα can be also detected in cells co-transfected with plasmids encoding histidinylated versions of ubiquitin with or without vectors expressing IκBα WT to purify exogenous and endogenous ubiquitylated IκBα respectively (Figure 2D). Monoubiquitylated IκBα can also be easily reproduced *in vitro* using an ubiquitin mutant (Ub KO) where all reactive lysine residues have been changed to arginine (Figure 2E). However, monoubiquitylated IκBα cannot be immunoprecipitated with monoclonal or polyclonal IκBα antibodies, alone or combined in a TUBEs-IκBα immunoprecipitation procedure (Figure S1 and S2). Under these conditions monoubiquitylated IκBα is detected in the unbound fraction. Thus, the monoubiquitylated form of IκBα, found in organs and cell lines, shows a poor accessibility to IκBα immunoprecipitation but can be detected using denaturing gels followed by Western blot analysis.

### Monoubiquitylated IκBα is not sensitive to the TNFα-mediated degradation

**Figure 3:**
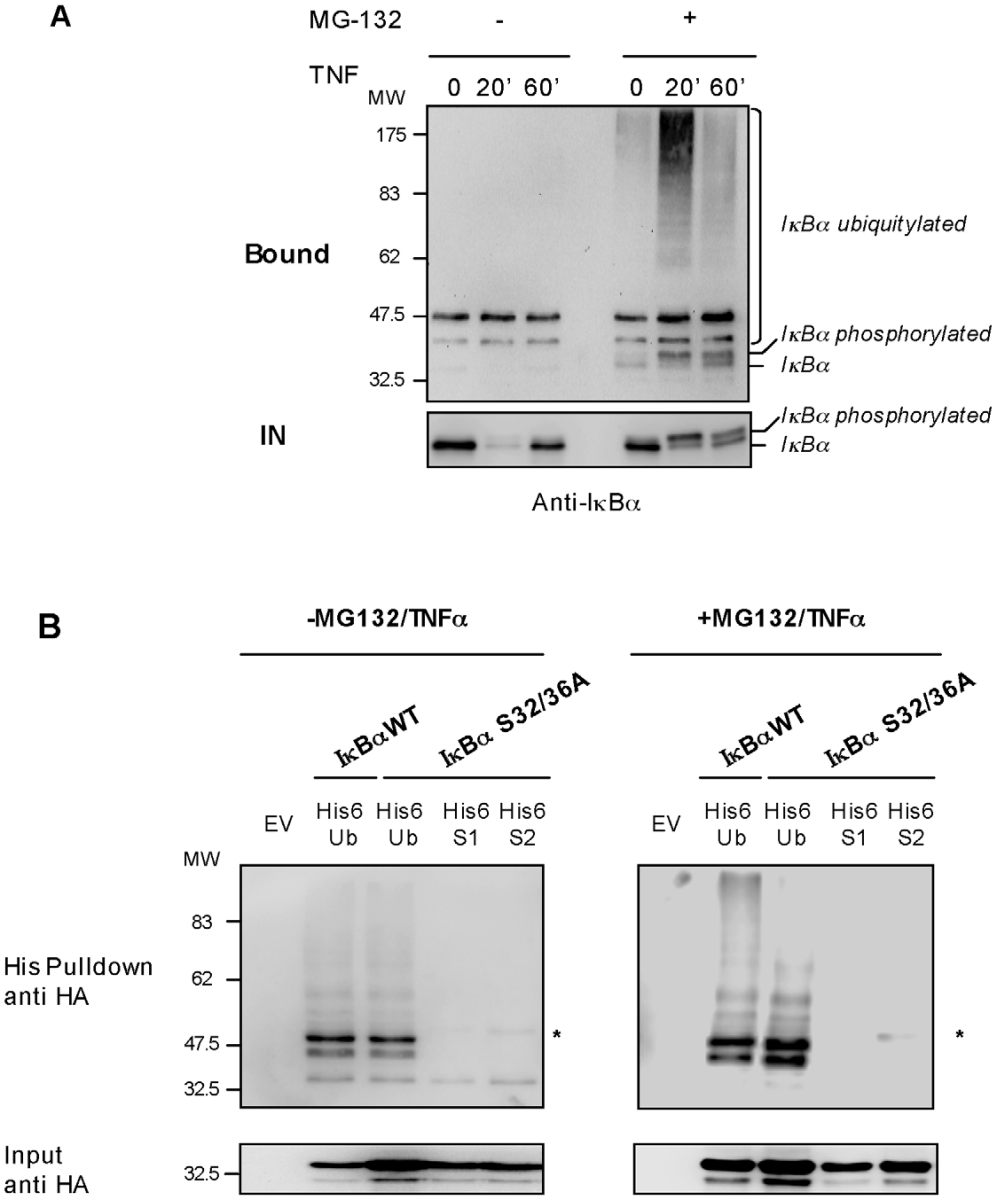
Monoubiquitylated IκBα is not sensitive to TNFα-mediated degradation. A) HEK293 cells were pre-treated or not 1 h with 20 µM of MG132 and stimulated with TNFα (10 ng/ml) for the indicated times. Cells were lysed in a buffer containing TUBE-hHR23A and bound proteins were analyzed by Western blot with the anti-IκBα antibody. B) HEK293 cells were transfected with the indicated plasmids, pre-treated or not 1 h with 20 µM MG132 and stimulated with TNFα (10 ng/ml). His_6_-ubiquitylated or SUMOylated proteins were purified using denaturing conditions and Ni^2+^ chromatography procedure. The SUMOylated form of IκBα is indicated by an *. EV: Empty vector.

To evaluate the susceptibility of the monoubiquitylated form of IκBα to be degraded by TNFα a TUBE-capture assay was performed in HEK293 cells treated or not with proteasome inhibitor MG132 (Figure 3). As expected, most IκBα is degraded after 20 minutes of TNFα-stimulation as it can be seen in the input (IN). This proteolytical process is blocked in the presence of MG132 where IκBα is accumulated as hyperphosphorylated form. The analysis of the TUBE-captured material shows that monoubiquitylated IκBα remain very stable after 20 or 60 minutes of TNFα stimulation even in the presence of proteasome inhibitor (Figure 3A). Interestingly, the capacity of the TUBE-hHR23 to capture monoubiquitylated and polyubiquitylated forms of IκBα is not compromised when the proteasome activity is inhibited. These results suggest that the monoubiquitylated form of IκBα is not destabilized by the induction with TNFα but it is slightly accumulated after treatment with MG132 (Figure 3A). To evaluate the role of the serines 32/36 phosphorylation on the accumulation of monoubiquitylated form of IκBα, a mutant S32/36A was transfected into HEK293 cells. In the absence of TNFα stimulation and MG132, modified forms of IκBα were captured using His6-Ubiquitin, His6-SUMO-1 or His6-SUMO-2 and nickel beads chromatography. Our results confirm that the monoubiquitylation of IκBα is not dependent of the phosphorylation of serines 32 and 36 (Figure 3B). MonoSUMOylation with SUMO-2 and SUMO-1 (only visible on long exposures, data not shown) are also independent of this signaling pathway. In contrast, high molecular weight forms can not be seen on the S32/36A IκBα mutant after TNFα stimulation in a situation where polyubiquitylated IκBα WT is well accumulated (Figure 3B).

### Extended half-life of monoubiquitylated IκBα

**Figure 4:**
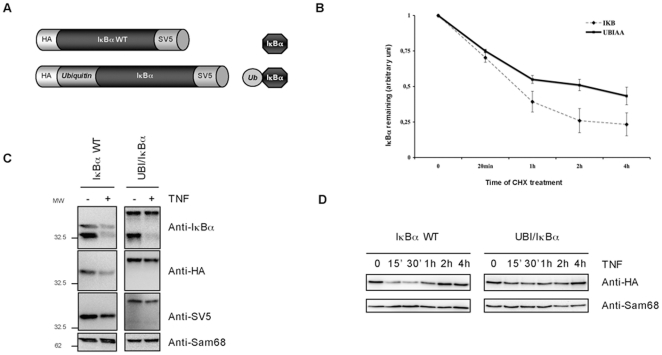
Stability of ubiquitin-IκBα fusion protein. A) Ubiquitin-IκBα fusion was generated with an N-terminal HA and C-terminal V5 epitopes. B) HEK293 cells transfected with IκBα WT and ubiquitin-IκBα fusion were treated with 50 µg/ml CHX for the indicated times. The graph corresponds to the mean of three independent experiments. C) IκBα WT and ubiquitin-IκBα fusion were expressed in HEK293 and stimulated for 20 minutes with 10 ng/ml of TNFα. D) IκBα WT and ubiquitin- IκBα fusionwere expressed in HEK293 cells and stimulated with 10 ng/ml of TNFα for the indicated times.

The analysis of a subpopulation of IκBα molecules and in particular its impact on NF-κB activity is difficult to achieve if mixed populations of IκBα molecules are present. There is no available method of purification able to isolate unmodified or ubiquitylated IκBα molecules with homogeneous characteristics. For this reason, to further understand the role of monoubiquitylated form of IκBα, an ubiquitin-IκBα fusion protein was generated (Figure 4A). This approach has been largely used as it can be judged in the literature especially to study the role of ubiquitin and ubiquitin-like proteins in the regulation of protein localization and function [15]. The ubiquitin-IκBα fusion has been optimized to resist to the action of DUBs by introducing, at the C-terminal of ubiquitin, a double alanine (AA) instead of the double glycine (GG). To avoid additional attachment of moieties at the N-terminus of IκBα, lysine 21 and 22 were mutated to alanine (KK to AA). Attachment of ubiquitin at a single N-terminus lysine acceptor of IκBα provide similar stability effects [16], [17]. Ubiquitin-IκBα fusion protein shows similar sub-cellular distribution than IκBα WT (Figure S3). When expressed in HEK293 and HeLa cells, ubiquitin-IκBα fusion protein showed an extended half-life compared to IκBα WT (Figure 4B and data not shown). The effect of a single ubiquitin moiety on IκBα stability is also reflected after signal-mediated stimulation, as this ubiquitin-IκBα fusion shows resistance to TNFα induced degradation (Figure 4C). A kinetic of degradation was performed to confirm that the observed resistance was not due to a delay in TNFα-induced IκBα degradation (Figure 4D). Proteolytical defects are not due to the mutation of the Lysine 21 or/and 22 since its presence in other ubiquitin-IκBα fusions provides similar results (data not shown). Furthermore, these effects appear to be specific of ubiquitin, as fusions containing other molecules from the ubiquitin family do not provide the same results (data not shown). Thus, from these results we conclude that the attachment of a single ubiquitin moiety extends the half-life of the ubiquitin-IκBα fusion and perfectly reproduce the stability after TNFα-stimulation observed with the endogenous monoubiquitylated form of IκBα.

### Phosphorylation defects of monoubiquitylated IκBα

**Figure 5:**
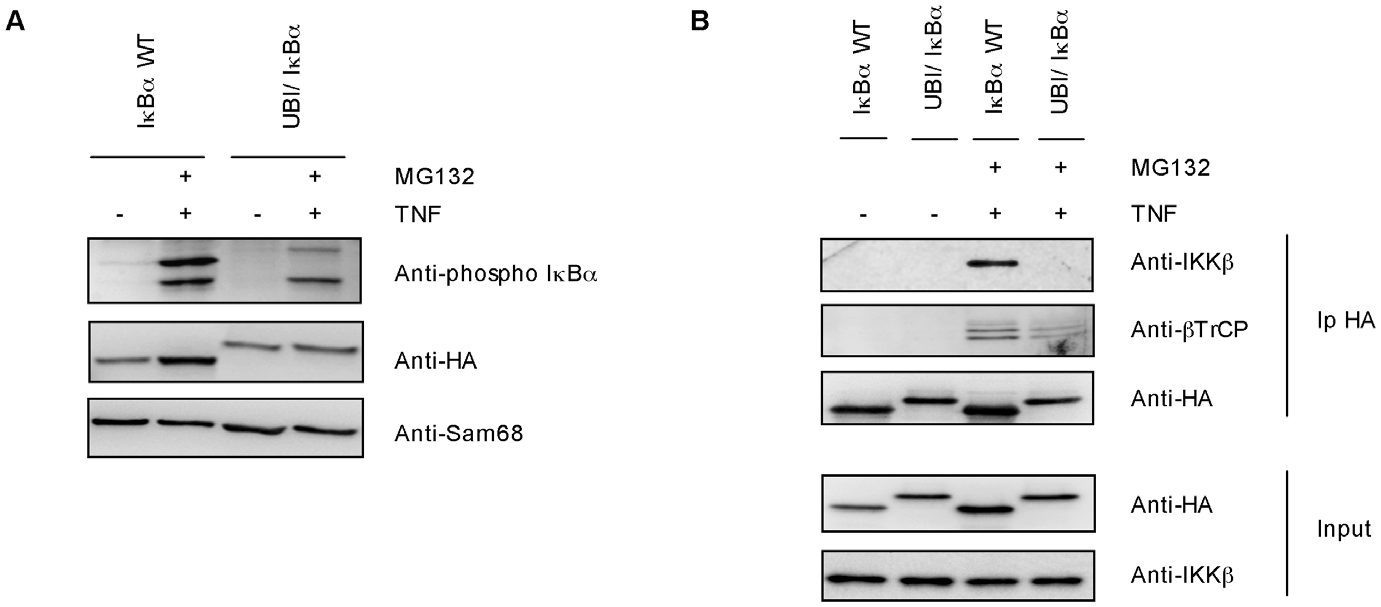
Phosphorylation defect of ubiquitin-IκBα fusion. A) IκBα WT and ubiquitin-IκBα fusion were expressed in HEK293 cells and pretreated or not with 20 µM of MG132 for 1 hour before being stimulated with 10 ng/ml of TNFα for 20 minutes. Sam68 was used as a charge control B) Ubiquitin-IκBα fusion deficiently binds endogenous IKKβ kinase and the βTrCP. IκBα WT and ubiquitin-IκBα fusion were expressed in HEK293 cells, pretreated or not with 20 µM of MG132 for 1 hour and stimulated with 10 ng/ml of TNFα for 20 minutes. Cell lysates were immunoprecipitated with HA antibody and analyzed by Western-blot using indicated antibodies.

In order to understand the molecular origin of ubiquitin-IκBα fusion stability, its capacity to be phosphorylated after TNFα stimulation was investigated. We could observe a reproducible reduction of ubiquitin-IκBα fusion phosphorylation when compared to IκBα WT (Figure 5A). This is mainly due to the incapacity of ubiquitin-IκBα fusion protein to efficiently bind IKKβ compared to IκBα WT in TNFα-stimulated HEK293 cells (Figure 5B). Experimental data demonstrate that the exogenously expressed ubiquitin-IκBα fusion also fails to efficiently interact with βTrCP compared to IκBα WT under the same experimental conditions. Altogether our results clearly indicate that ubiquitin-IκBα fusion but not IκBα WT shows defects in the interaction with critical molecules of the signaling pathway including IKKβ and βTrCP, thus explaining at least in part, its resistance to proteolysis (Figure 5B).

### Monoubiquitylated IκBα negatively affects NF-κB activity

**Figure 6:**
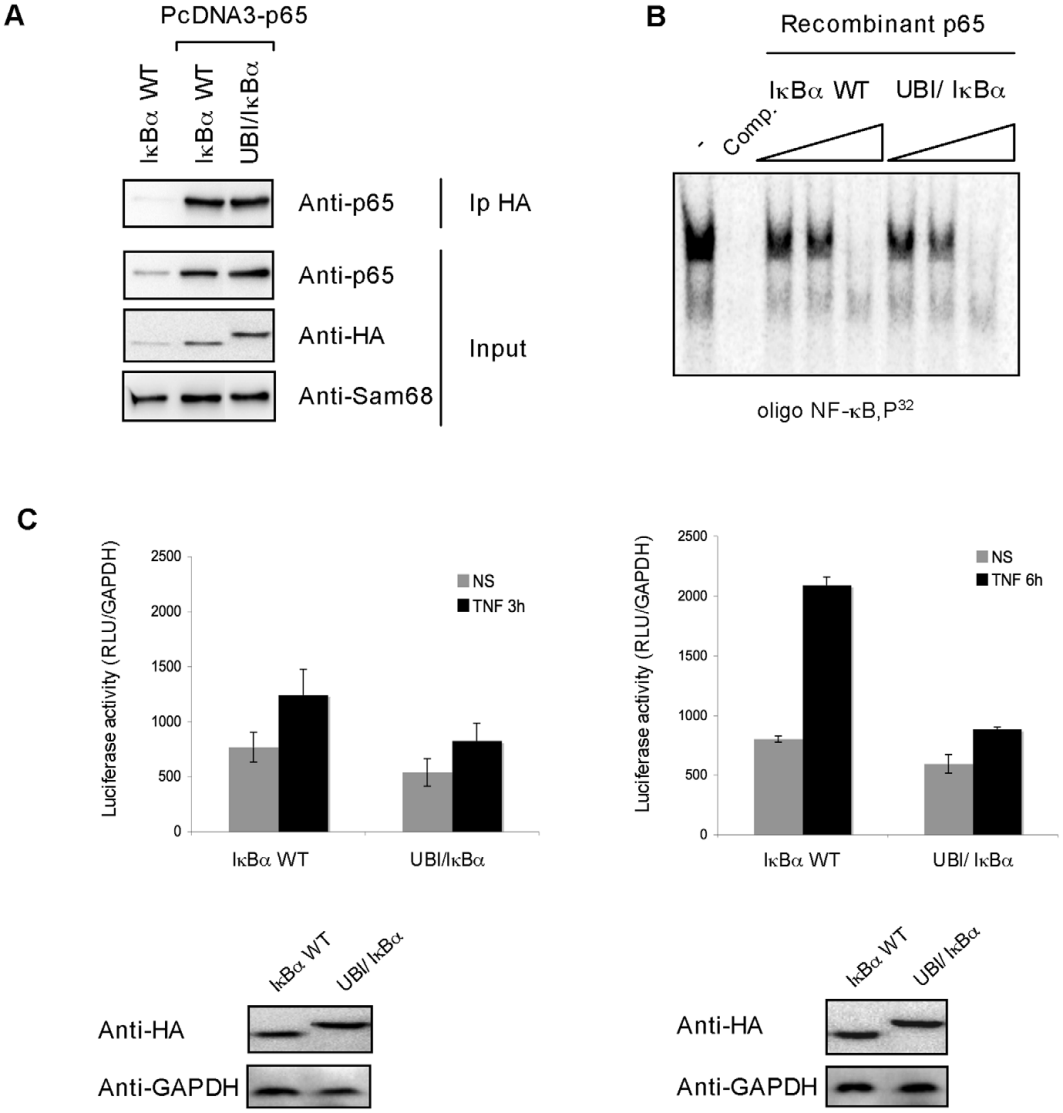
Ubiquitin-IκBα fusion negatively affects TNF-induced NF-κB activity. A) Ubiquitin-IκBα fusion protein has the same capacity as unmodified IκBα to bind to NF-κB. HEK293 cells were transfected with the indicated plasmids. Lysates were submitted to anti-HA immunoprecipitation and Western blotted with the indicated antibodies B) Ubiquitin-IκBα fusion protein inhibits NF-κB/DNA binding as well as unmodified IκBα. Different amount of recombinant IκBα fusions proteins (1: 0,05 µl, 2: 0,1 µl and 3: 0,5 µl) and p65 were incubated with a radioactive labeled NF-κB probe for EMSA studies [12]. Comp.: competition with a 100-fold excess of the same unlabeled oligonucleotide added to the binding assay before the ^32^P-labeled probe. The graph corresponds to the mean of three independent experiments. C) IκBα KO fibroblasts were co-transfected with IκBα WT or ubiquitin- IκBα fusion expressing plasmid and a NF-κB-luciferase reporter (3 EnhConA-Luc). Luciferase activity was measured as previously described [17]. The graph corresponds to the mean of three independent experiments.

**Figure 7:**
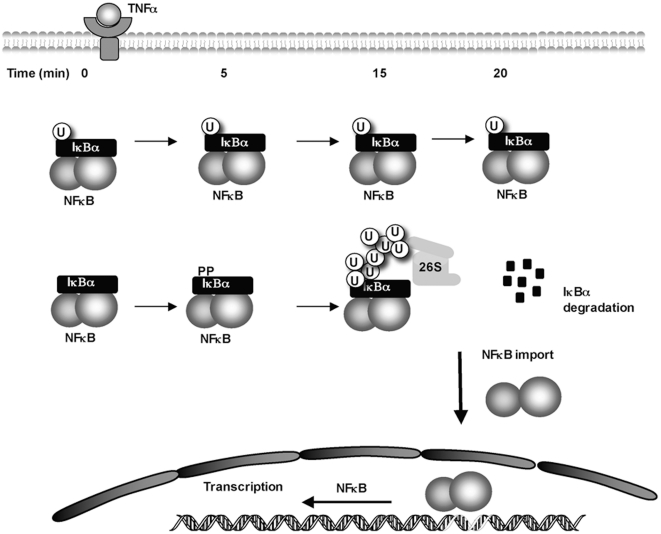
Integrated view of the time-dependent contribution of monoubiquitylated versus non-modified form of IκBα in the control of its proteasomal degradation and regulation of NF-κB activity.

To investigate the effect of ubiquitin-IκBα fusion on the NF-κB activity, first we explored its capacity to bind NF-κB and to inhibit NF-κB/DNA binding. Interaction with the NF-κB subunit p65 was tested in HEK293 cells expressing or not exogenous p65 and ubiquitin-IκBα or IκBα WT as indicated in Figure 6A. Our results clearly indicate that p65 co-immunoprecipitates equally well with both ubiquitin-IκBα and IκBα WT. To explore the capacity of ubiquitin-IκBα fusion to inhibit NF-κB/DNA binding, electrophoretic mobility shift assay (EMSA) were performed using increasing concentrations of both ubiquitin-IκBα and IκBα WT. As observed in Figure 6B, the capacity of ubiquitin-IκBα fusion to inhibit NF-κB/DNA interaction is similar to the one of IκBα WT. To further explore the effect of ubiquitin-IκBα on the NF-κB activity after TNFα stimulation, luciferase reporter assays were set up. To avoid interferences with endogenous IκBα MEF coming from IκBα KO mice were employed. The inhibitory effect of ubiquitin-IκBα fusion became statistically significant after 6 hours of TNFα stimulation (Figure 6C). Thus, our results indicate that in the presence of ubiquitin-IκBα fusion, activation of NF-κB is negatively regulated after TNFα stimulation. Altogether, our results suggest the existence of distinct populations of IκBα molecules among which monoubiquitylated IκBα offers resistance to TNFα mediated degradation preserving a dormant pool of NF-κB that is different to the one activated through the activation of this signaling pathway (Figure 7).

## Discussion

While polyubiquitylation of proteins has been associated to the regulation of signaling cascades or protein degradation by the Ubiquitin Proteasome System, monoubiquitylation or multiple monoubiquitylation have diverse non-catabolic functions [18]. Major technical problems to separate the ubiquitylated pool of endogenous proteins from unmodified ones justify our limited knowledge of the post-modification events. The broad distribution of monoubiquitylated IκBα in multiple organs and cell lines underlines the *in vivo* importance of this pool of IκBα. Endogenous monoubiquitylated IκBα is stable after TNFα stimulation and does coexist with polyubiquitylated IκBα under the same conditions. Therefore monoubiquitylated IκBα does not appear to be a precursor of polyubiquitylated forms of this inhibitor molecule, although a dynamic equilibrium between these populations cannot be excluded. Monoubiquitylated IκBα accumulated after proteasome inhibitor and TNFα treatment can be the result of proofreading mechanism acting on polyubiquitylated IκBα. With the help of a DUB-resistant ubiquitin-IκBα fusion, results presented here show that monoubiquitylated IκBα has an impact on basal and TNFα-induced NF-κB transcription. It remains to be investigated the nature of the stimuli (if any) able to drive an efficient proteolysis of monoubiquitylated IκBα. In a similar way SUMO-1 was reported to regulate IκBα stability and NF-κB transcription [10]. However it is unclear if both IκBα pools cooperate with each other to regulate basal and/or signal mediated turnover. Several lines of evidence suggest that monoubiquitylated IκBα might adopt a structured/protected conformation. The first evidence is the difficulty to pull down endogenous monoubiquitylated IκBα with various monoclonal and polyclonal antibodies. Second, the ubiquitin-IκBα fusion is not efficiently recognized by the IKKβ subunit resulting in a limited phosphorylation and binding to βTrCP. Finally, the impact of the fused ubiquitin on the IκBα resistance to TNFα-induced proteolysis, suggest reduced capacity to interact/get access to the proteasome. Attachment of monoubiquitin onto IκBα perhaps occludes IKK binding sites or creates molecular interference with this kinase. Under these circumstances, βTrCP might have difficulties to polyubiquitylate poorly phosphorylated IκBα. The unstructured extremities of IκBα favor the ubiquitin-independent proteasomal degradation of this molecule [19] justifying the necessity to generate a stable pool of monoubiquitylated IκBα. If there is an ubiquitin-protein ligase different than the SCF-βTrCP complex, it has to be proven. However, one has to keep in mind that E3-independent monoubiquitylation has been reported for proteins containing ubiquitin-binding domains [20]. Recently, monoubiquitylation of Rpn10 subunit of the proteasome has been shown to adopt a closed conformation due to the intra-molecular interaction with its ubiquitin interacting motif or UIM [21]. In the case of Rpn10, monoubiquitylation affects presentation of ubiquitylated proteins to the proteasome. However, if this occluding mechanism exists for monoubiquitylated IκBα there is no evidence of an ubiquitin-binding domain present on this inhibitor. Nonetheless, the active molecular dynamics reported for the NF-κB system even under basal conditions [22] [23] [24] justifies the existence of monoubiquitylated IκBα as a cellular reservoir to regulate basal as well as signal-mediated NF-κB activity. Knowing the resistance to proteolysis observed for monoubiquitylated IκBα, one can speculate that an artificial increase of this pool could let to a better control of immune and/or pro-inflammatory responses found in organisms that have been exposed to multiple and/or sequential stimuli activating NF-κB. Future work will elucidate the role of the different populations of IκBα in the optimal control of this critical transcription factor.

## Materials and Methods

### Animals

#### Ethics Statement

Experiments were approved by the respective institutional committees for animal care and handling.

Adult male Sprague–Dawley rats were deeply anesthetized with chloral hydrate and some tissues and organs were extracted. These samples were washed with cold PBS, immediately frozen in liquid nitrogen and stored at −80°C.

### Cell cultures

HEK293 and HeLa (ATCC) were grown in DMEM (Gibco); Jurkat cells (ATCC) in RPMI (Gibco), all supplemented with 10% FBS and antibiotics. HEK293 and HeLa were transfected using lipofectamine (Invitrogen). For measurement of transcriptional activity, MEF null IκBα (kindly given by David Baltimore) were co-transfected with a NF-κB-luciferase reporter plasmid (3-EnhConA) and plasmids expressing IκBα WT or the ubiquitin-IκBα fusion. Luciferase activity was measured as previously described [17]. To analyze the half-life of the different proteins, cells were treated with 50 µg/ml of cycloheximide (Sigma) during the indicated times. For stability experiments, cells were treated for 1 hour with 20 µM of MG132 (Calbiochem), stimulated for 30 minutes or the indicated times with 10 ng/ml of TNFα(R&D Systems). P65, IKKβ and βTrCP co-immunoprecipitation experiments were performed using Protein-G cross-linked with the HA antibody to immunoprecipitate exogenous IκBα WT or ubiquitin-IκBα fusion protein. In all cases, cells were lysed for 15 minutes on ice in 50 mM sodium fluoride, 5 mM tetra-sodium pyrophosphate, 10 mM beta-glyceropyrophosphate, 1% Igepal CA-630, 2 mM EDTA, 20 mM Na_2_HPO_4_, 20 mM NaH_2_PO_4_, 1 mM Pefablock, 1.2 mg/ml Complete protease inhibitor cocktail (Roche).

### PCR and cloning

Ubiquitin gene (accession numbers CAA44911) was used to generate IκBα fusion and cloned into BamHI/Not1 restriction sites of pCDNA3. The C-terminal glycine residues (GG) of ubiquitin were changed to alanine (AA) and lysine 21 and 22 of IκBα were mutated to alanine to avoid respectively the action of DUBs and additional attachment of moieties at the N-terminus of IκBα, using the following oligonucleotides: 5′-ctc cgt ctt aga gct gcg gag cgg cta ctg gac gac-3′ and 5′-gtc gtc cag tag ccg ctc cgc agc tct aag acg gag-3′. His6-Ubiquitin construct has been previously reported [13].

### Purification of ubiquitylated proteins

Frozen tissues were triturated in liquid nitrogen and recovered in the previously reported lysis buffers [14], containing 200 µg of TUBEs-HR23A (T) (Life Sensors) or Glutathione S-transferase (GST) (C). Lysates were clarified by cold centrifugation, and added to glutathione agarose beads (Sigma). Glutathione beads were eluted and bound material was submitted to Western-blot analysis or to IκBα (Cell Signaling) and ubiquitin (FK2, ENZO) immunoprecipitations. His6-ubiquitylated proteins were purified using denaturing conditions and Ni^2+^ chromatography as previously described [13].

### Western blotting

Immunodetections were performed with the following primary antibodies: mouse monoclonals SV5 (Serotec); HA (Covance); Ubiquitin P4D1 (Santa Cruz Biotechnology Inc), FK2 (ENZO); IκBα (Cell Signaling), IκBα 10B (kindly provided by RT Hay) and anti-phospho-IκBα (Cell Signaling) antibodies; rabbit polyclonals IκBα (C21) (Santa Cruz Biotechnology); p65 (Santa Cruz Biotechnology); IKKβ (Cell Signaling) and Sam68 (Santa Cruz Biotechnology) antibodies and goat polyclonal antibody βTrCP (Santa Cruz Biotechnology).

### *In vitro* ubiquitylation assays

*In vitro* transcribed/translated IκBα (^35^S-Met-labelled or not when indicated) were incubated in a 15 µl reaction including an ATP regenerating system [25 mM Tris pH 7.5, 5 mM MgCl2, 2 mM ATP, 10 mM creatine phosphate (Sigma), 5 mM NaCl_2_, 3.5 U/ml of creatine kinase (Sigma) and 0.6 U/ml of inorganic pyrophosphatase (Sigma)], 10 µg of ubiquitin mutant (Ub KO) where all reactive lysine residues have been changed to arginine ubiquitin, 10 ng human E1 (Biomol), 500 ng UbcH5b (Biomol). After incubation at 30°C for 120 min the reaction was stopped with SDS Laemmli buffer containing β-mercaptoethanol, samples were fractionated by SDS-PAGE and the dried gels analysed by phosphorimaging.

### Electrophoretic Mobility Shift Assays (EMSA)

Reactions were prepared in binding buffer containing 25 mM HEPES, 1 mM EDTA, 3.5 mM spermidine, 6 mM MgCl2, 100 mM NaCl, 0.15% Nonidet P-40, 10% glycerol, 10 mM Dithiothreitol, 1 mg/ml bovine serum albumin and 0.05 mg Poly dAT/dGC, different amount of IκBα fusion proteins (1: 0,05 µl, 2: 0,1 µl and 3: 0,5 µl) and recombinant protein p65 and incubated at room temperature for 20 minutes. Finally, 10000 cpm of ^32^P-radiolabelled (polynucleotide kinase, Biolabs) double strand oligonucleotide probe containing the NF-κB binding site motif from the HIV type 1 enhancer (5′-CTA GAC GGG GAT TTC CGA GAG GT-3′) was added and the mixture was incubated at room temperature for 20 minutes. After electrophoresis, gels were dried and exposed to Amersham Hyperfilm MP at −70·C. Specific binding was checked by competition with a 100-fold excess of the same unlabeled oligonucleotide added to the binding assay before the ^32^P-labeled probe.

## Supporting Information

Figure S1 Immunoprecipitation using IκBα antibodies fail to pull down monoubiquitylated IκBα. HEK293 cells were treated or not for 1 hour with 20 µM of MG-132, lysed in the properly lysis buffer for 20 minutes, centrifuged and the supernatant was incubated with cross-linked anti- IκBα (10B) antibody for 2 hours. After incubation the samples were centrifuged, washed and prepared for Western blot analysis using IκBα antibody (Cell Signaling).
(TIF)

Figure S2 TUBE-captured monoubiquitylated IκBα fails to be immunoprecipitated using specific IκBα antibodies. HEK293 cells were treated or not, 1 hour with 20 µM of MG-132 and lysed in a buffer containing 100 µg of TUBE-HR23A or GST proteins. After lysis, samples were centrifuged and clarified supernatant incubated for 2 hours in the presence of glutathione agarose beads. Eluted samples were incubated for 2 hours with protein A cross-linked antibody anti-IκBα 10B or anti-IκBα C21 antibody (not shown). After incubation, samples were washed and prepared for Western blot analysis using IκBα antibody (Cell Signaling).
(TIF)

Figure S3 IκBαWT and ubiquitin-IκBα fusion were expressed in HEK293 cells, and processed for immunostaining with anti-SV5 or anti-HA antibodies.
(TIF)
