# Supplementary figures and images for: Role of Monoubiquitylation on the Control of IκBα Degradation and NF-κB Activity
Source: PLoS One. 2011 Oct 12;6(10):e25397. doi: 10.1371/journal.pone.0025397 (PMC3192046; doi:10.1371/journal.pone.0025397)

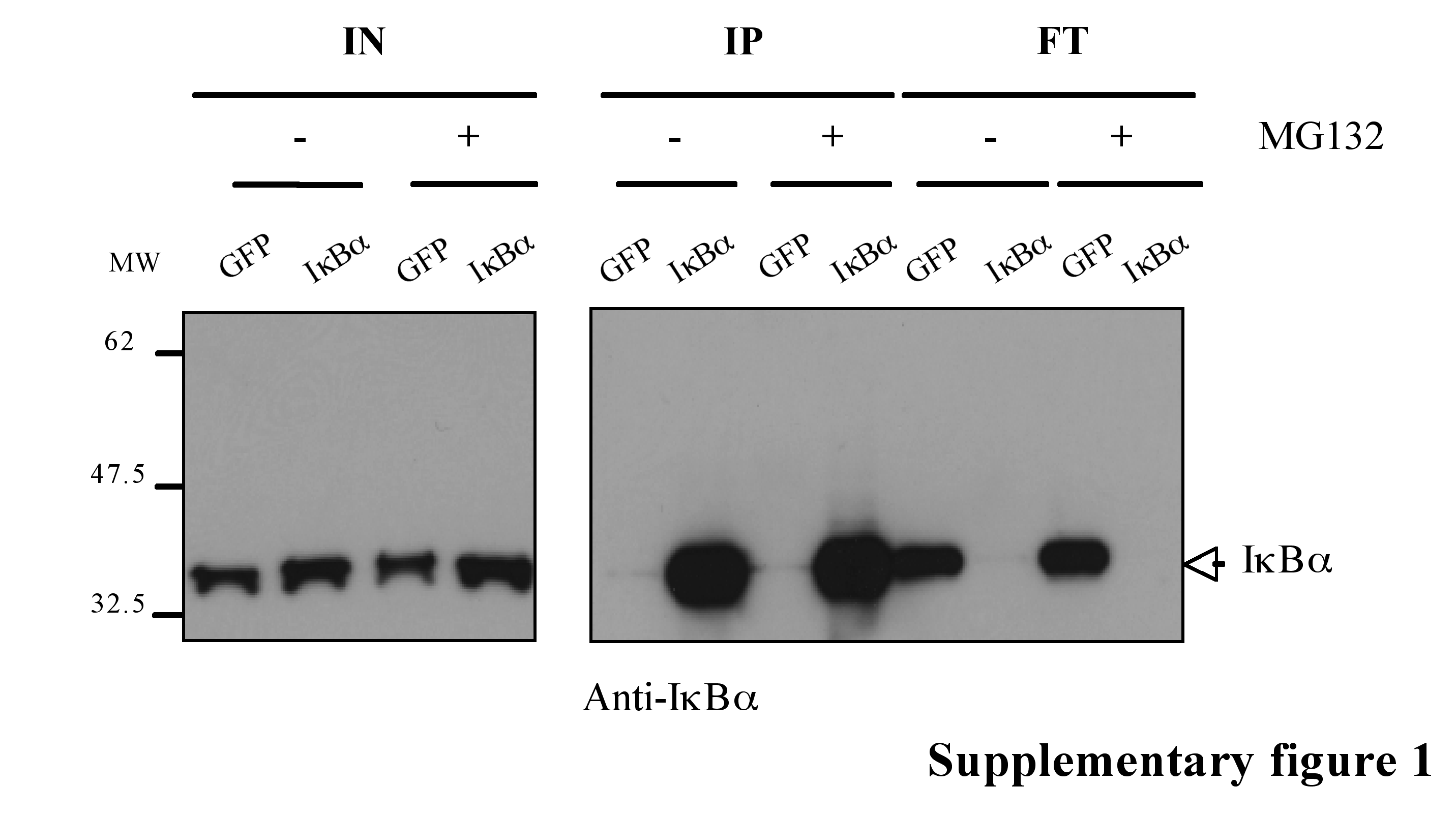

Supplement: Figure S1 — Immunoprecipitation using IκBα antibodies fail to pull down monoubiquitylated IκBα. HEK293 cells were treated or not for 1 hour with 20 µM of MG-132, lysed in the properly lysis buffer for 20 minutes, centrifuged and the supernatant was incubated with cross-linked anti- IκBα (10B) antibody for 2 hours. After incubation the samples were centrifuged, washed and prepared for Western blot analysis using IκBα antibody (Cell Signaling). (TIF) [file pone.0025397.s001.tif]

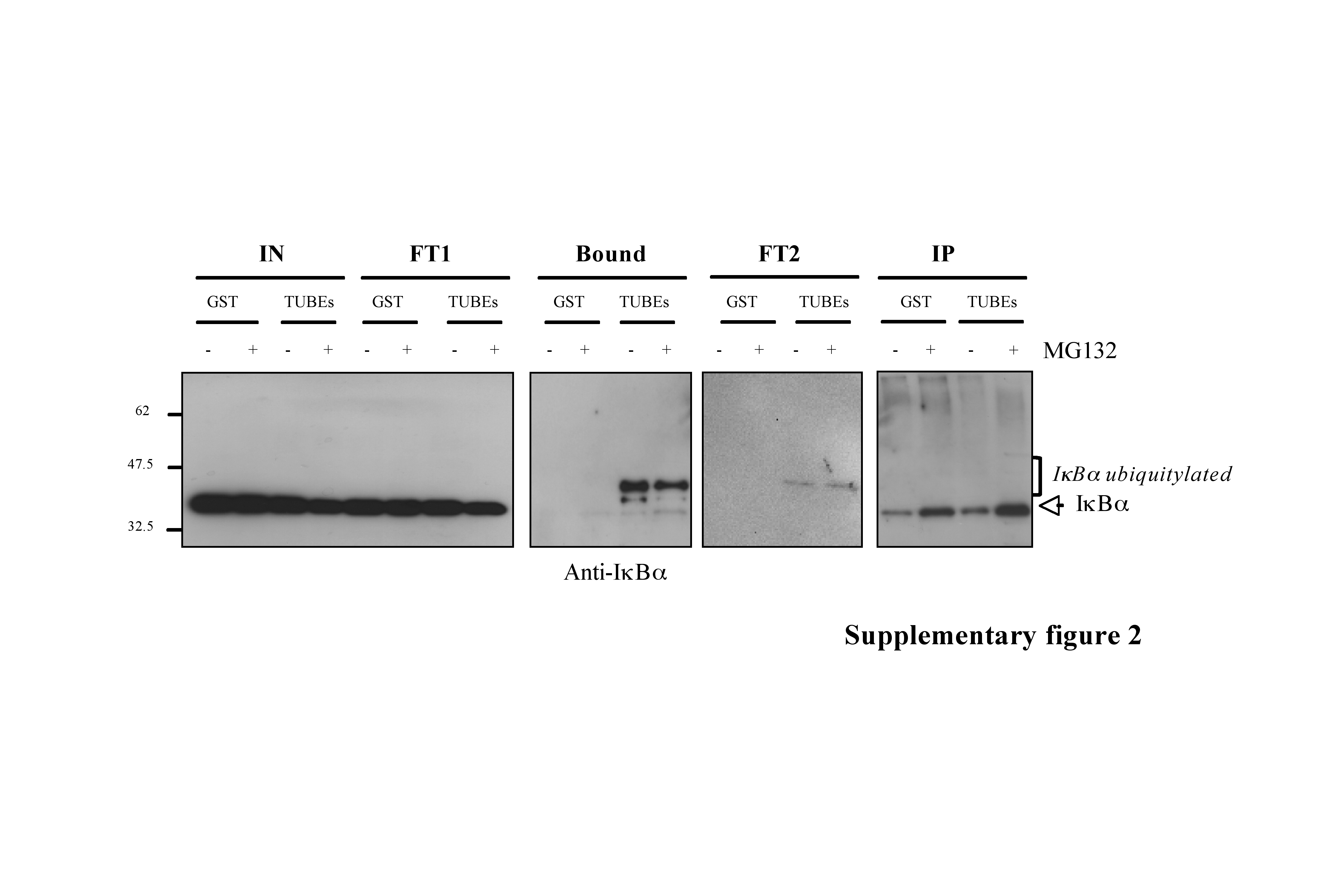

Supplement: Figure S2 — TUBE-captured monoubiquitylated IκBα fails to be immunoprecipitated using specific IκBα antibodies. HEK293 cells were treated or not, 1 hour with 20 µM of MG-132 and lysed in a buffer containing 100 µg of TUBE-HR23A or GST proteins. After lysis, samples were centrifuged and clarified supernatant incubated for 2 hours in the presence of glutathione agarose beads. Eluted samples were incubated for 2 hours with protein A cross-linked antibody anti-IκBα 10B or anti-IκBα C21 antibody (not shown). After incubation, samples were washed and prepared for Western blot analysis using IκBα antibody (Cell Signaling). (TIF) [file pone.0025397.s002.tif]

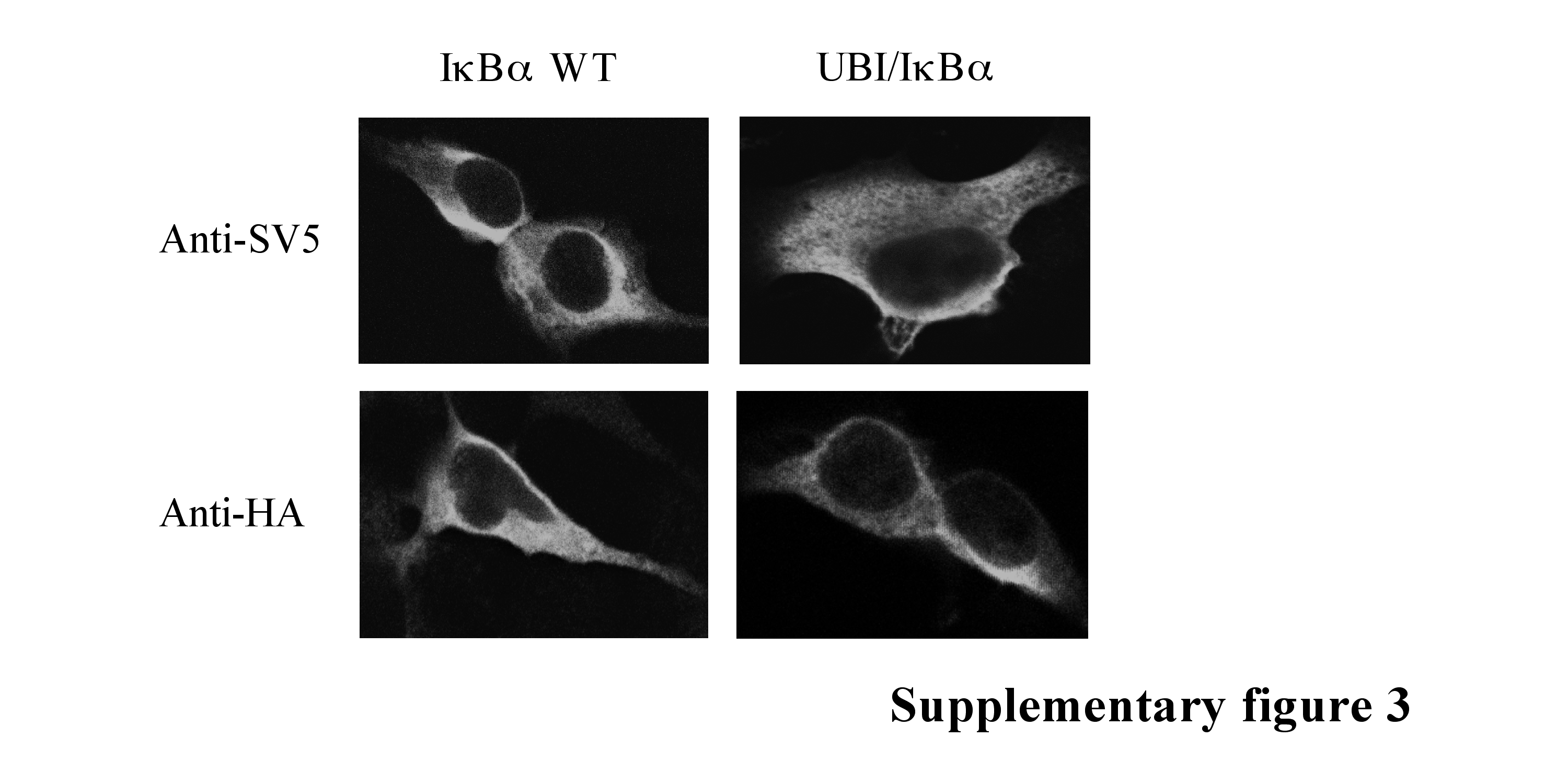

Supplement: Figure S3 — IκBαWT and ubiquitin-IκBα fusion were expressed in HEK293 cells, and processed for immunostaining with anti-SV5 or anti-HA antibodies. (TIF) [file pone.0025397.s003.tif]
